# Synergistic Cytotoxic Effect of Gold Nanoparticles and 5-Aminolevulinic Acid-Mediated Photodynamic Therapy against Skin Cancer Cells

**Published:** 2014-09

**Authors:** Mahnaz Hadizadeh, Mohsen Fateh

**Affiliations:** 1 Biotechnology Center, Iranian Research Organization for Science and Technology, Tehran, Iran;; 2 Medical Laser Research Center, Iranian Center for Medical lasers, Academic Center for Education, Culture and Research, Tehran, Iran

**Keywords:** Photochemotherapy, Aminolevulinic acid, Nanoparticles

## Abstract

**Background: **Photodynamic therapy (PDT) is a promising therapeutic modality for the treatment of cancer and other diseases. In this study, the epidermoid carcinoma cell line A431 and the normal fibroblasts were used to investigate whether gold nanoparticles (GNPs) can induce an increase in cell death during PDT using 5-aminolevulinic acid (5-ALA) as a photosensitizer.

**Methods:** Human fibroblast and A431 cells were grown in 96-well plates. The effect of GNPs on the efficacy of 5-ALA-mediated PDT (5-ALA-PDT) was evaluated by comparing the effect of 5-ALA with GNPs to the effect of 5-ALA alone. Cell viability was determined by the methyl- tetrazolium assay.

**Results: **Dark toxicity experiments showed that 5-ALA at concentrations 0.5, 1 and 2 mM had no significant effect on cell viability of both cell lines. However, treatment of cells with 5-ALA (0.5 to 2 mM) and light dose of 25 Jcm^-2^ led to 5-10% and 31-42% decrease in cell viability of fibroblast and A431 cells respectively. The data also shows that GNPs in both the absence and the presence of light, results in a dose-dependent decrease in cell viability of both cell lines. However, the sensitivity of cancer cells to GNPs at concentrations more than 24 μg/ml was approximately 2.5- to 4-fold greater than healthy cells. Furthermore, data indicates that 5-ALA in combination with GNPs results in a synergistic reduction in viability of A431 cells.

**Conclusion: **In summary, the findings of this study suggest that concomitant treatment with 5-ALA and GNPs may be useful in enhancing the effect of 5-ALA-PDT.

## Introduction

Recently, in addition to conventional cancer treatments including surgery, chemotherapy and radiation therapy, new therapeutic modalities such as gene therapy, photodynamic therapy (PDT) and some targeted therapies including the use of monoclonal antibodies, anti-angiogenesis agents and growth factor inhibitors have been found useful in treating some types of cancers.^1^^-^^4^

PDT is known as a promising treatment for the management of cancer and several non-cancerous diseases that are generally characterized by overgrowth of abnormal cells.^5^^,^^6^ This form of therapy is based on applying a light-sensitive compound called photosensitizer with visible light at specific wavelength to excite the photosensitizer that preferentially accumulated in the diseased tissue. Following the activation of photosensitizer within cancer cells, reactive oxygen species (ROS) and other radicals produced by photochemical reactions result in the oxidative damage to intracellular macromolecules and death of cancer cells.^7^ The tumor cell death in PDT is induced via apoptosis, necrosis and autophagy, depending on cell type, light irradiation dose, photosensitizer concentration and its subcellular localization.^8^^,^^9^ A large number of compounds are used as photosensitizer in PDT but only photofrin (porfimer sodium), levulan (5-ALA) and metvix (methyl aminolevulinate) have received approval from the U.S. Food and Drug Administration (FDA) for PDT in treating certain types of cancer and other diseases.^10^

Although 5-ALA is not a photosensitizer, it is a metabolic precursor in the heme biosynthesis pathway. Hemoconcentration regulates the level of 5-ALA in cells. However, when cells are exposed to excess exogenous 5-ALA as a drug, the negative feedback control mechanism of 5-ALA synthesis is bypassed, leading to protoporphyrin IX accumulation in the mitochondria of malignant tissues where ferrochelatase enzyme is absent. Protoporphyrin IX , an immediate precursor of heme, can act as an effective photosensitizer for PDT.^11^^-^^13^ 5-ALA mediated photodynamic therapy (5-ALA-PDT) has been successfully used for the treatment of some skin disorders such as actinic keratosis,^14^ psoriasis^15^ and superficial basal cell carcinoma (BCC).^16^

Recently, due to their unique properties, the use of gold nanoparticles (GNPs) as a promising agents for cancer therapy have received great interests. As a result of thier small size, they can widely penetrate into a body, bind to many drugs and biomolecules and can be actively targeted towards cancer cells.^17^ Moreover, their non-ionizing radiation absorption features and unique surface plasmon resonance, allows them to be used in radiotherapy and photothermal therapy.^18^

In recent years, the use of non-toxic GNPs as photosensitizer carriers in cancer targeted PDT has also been considered.^19^^,^^20^ However, in the absence of any specific functionalization on the effects of PDT for the cancer treatment, little is known about the effect of biocompatible GNPs of different sizes.

The present study investigates whether 4 nm GNPs, not only as a carrier but also as a single agent, could induce an increase in cell death during PDT. For this purpose, A431 cells as a prototype of skin cancer cells and human fibroblasts as normal cells were used.

## Materials and Methods

*Chemicals*

Purchase of 5-ALA, trypan blue solution 0.4%, dimethyl sulfoxide (DMSO) and 3-(4,5-dimethylthiazol-2-yl)-2,5-diphenyltetrazoliumbromide (MTT) was carried out from Sigma-Aldrich (St Louis, MO, USA). Fetal bovine serum (FBS), phosphate buffered saline (PBS) and Non-phenol-Red RPMI 1640 medium were purchased from Dulbecco. All the other reagents were obtained from Merk.

*Synthesis of GNPs*

GNPs were prepared by the standard chemical reduction method of chloroauric acid (HAuCl_4_) by sodium borohydride (NaBH_4_) at room temperature. 12 ml of aqueous 0.5 mM hydrogen tetrachloroaurate(III) trihydrate stirred continuously with 0.5 ml of sodium citrate (Na_3_C_6_H_5_O_7_, 2H_2_O) 0.01 M for 15 min. Then 0.5 ml of ice-cold 0.1 M NaBH_4_ as a reducing agent was quickly added to the reaction mixture at once. Transmission electron microscopy (TEM) was utilized to confirm uniform creation of 4-5 nm GNPs.^21^

*Cell Culture*

The human fibroblasts and A431 cells were obtained from the Pasteur institute of Iran. Cells were grown in RPMI-1640 medium supplemented with 10% FBS, 100 Uml^-1^ penicillin, 0.1 mg ml^-1^ streptomycin and maintained at 37°C in a humidified atmosphere containing 5% CO_2_. 80% confluent cells were seeded in 96-well plates at a cell density of 15×10^3^ cell/well.^22^

*Dark and Light-Dependent Cytotoxicity*

To determine the cytotoxic effects of 5-ALA, GNPs and their combination, experiments were performed in four groups. In the first group, cells were incubated in medium alone (control group). In the second group, cells were incubated with different concentrations of 5-ALA for 4h. In the third group, cells were incubated with various concentrations of GNPs for 18h. In the final group, cells were incubated with various concentrations of GNPs for 18h and then 5-ALA was added in various concentrations to the cultured cells for additional 4h. Each concentration was tested in triplicate and the plates were incubated at 37°C.

The cells in each group were then either kept in the dark or illuminated with a GaAlInP diode laser device at 630 nm wavelength. The output power was 45 mW and the irradiation time was calculated to deliver a light dose of 25 Jcm^-2^. Dark- and photo-toxicity were assessed 24-h later by the MTT analysis.

*Cell Viability*

The viability of cells was determined by MTT colorimetric assay. At first, cells were incubated for 4h at 37°C with the thiazolyl blue tetrazolium bromide at a final concentration of 0.5mg/ml for 4h. The culture medium was replaced with 200 μl DMSO. The formazan crystals were dissolved by DMSO while shaking for 15 min. The absorbance was measured with an ELISA reader at 570 nm.^23^

*Statistical Analysis*

Data were expressed as means ± standard deviation (SD) for 3 independent experiments. Comparisons between means of groups were analyzed using one-way ANOVA and Tukey’s multiple-comparison tests. *P<0.05, **P<0.01 compared with respective control. Statistical differences were considered significant at *P<0.05.

## Results

*Dark- and Photo-Toxicity of GNPs*

**Figure 1:**
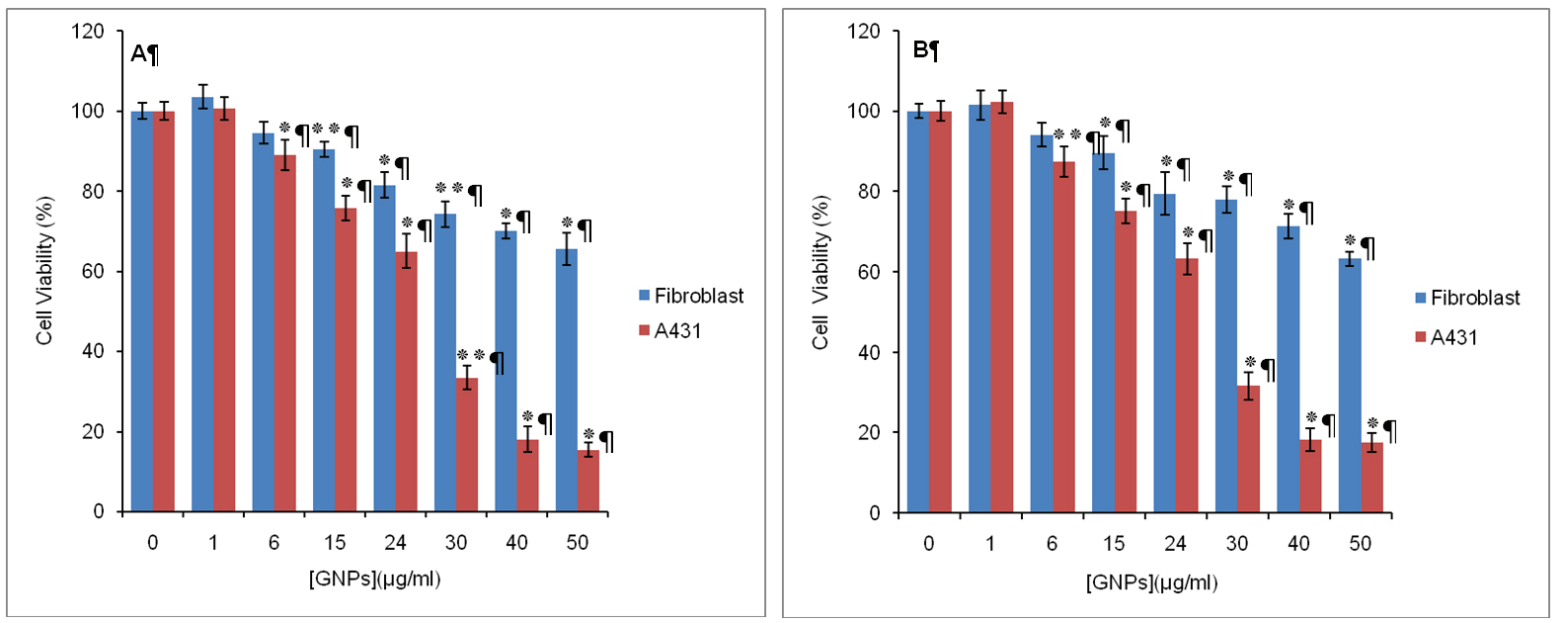
The effect of GNPs on cell viability in the human fibroblast and A431 cells. Cells (15,000) were treated with different concentrations of GNPs in the (A) dark or (B) light (λ=630 nm, 25 J/^2^) for 18 h. Cell viability is determined by MTT assay. Data are the mean±SD of at least three independent experiments (n=5). **P<0.01, *P<0.05 vs. control

As illustrated in figure 1A & 1B, GNPs, both the absence and the presence of light, resulted in a dose-dependent decrease in cell viability of A431 and fibroblast cells. However, the sensitivity of A431 cells to GNPs at concentrations more than 24 μg/ml was approximately 2.5- to 4-fold greater than fibroblast cells.

*Dark Cytotoxicity of 5-ALA*

**Figure 2:**
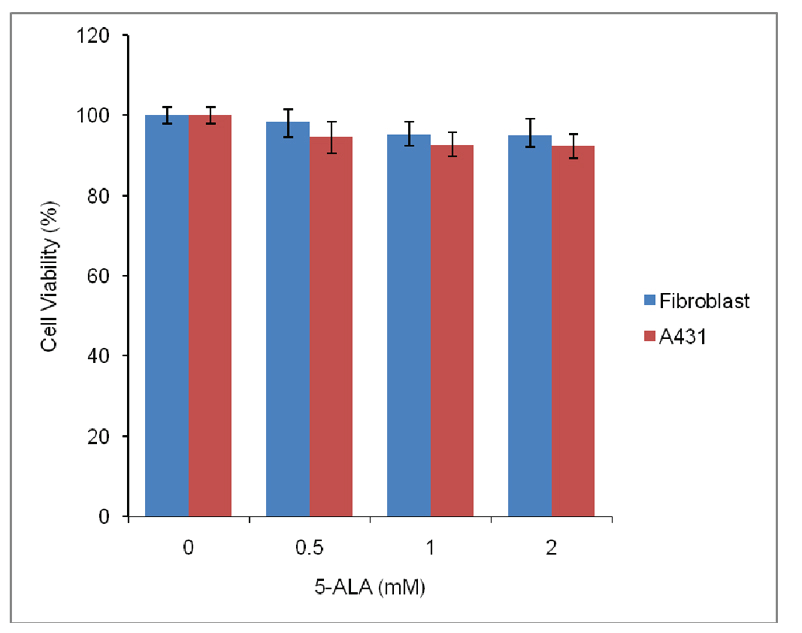
Cell viability (%) of human fibroblast and A431 cells (15,000), after 4 h incubation with different concentrations of 5-ALA in the absence of laser irradiation. Data are the mean±SD of at least three independent experiments (n=5).

Dark cytotoxicity of 5-ALA with different concentrations was evaluated in A431and fibroblast cells. According to figure 2, after 4 h incubation of cells in the dark with different concentration of 5-ALA, negligible cytotoxicity was detected (<10%) in both A431 and fibroblast cells.

*Dark Cytotoxicity of the Combination of 5-ALA and GNPs*

**Figure 3:**
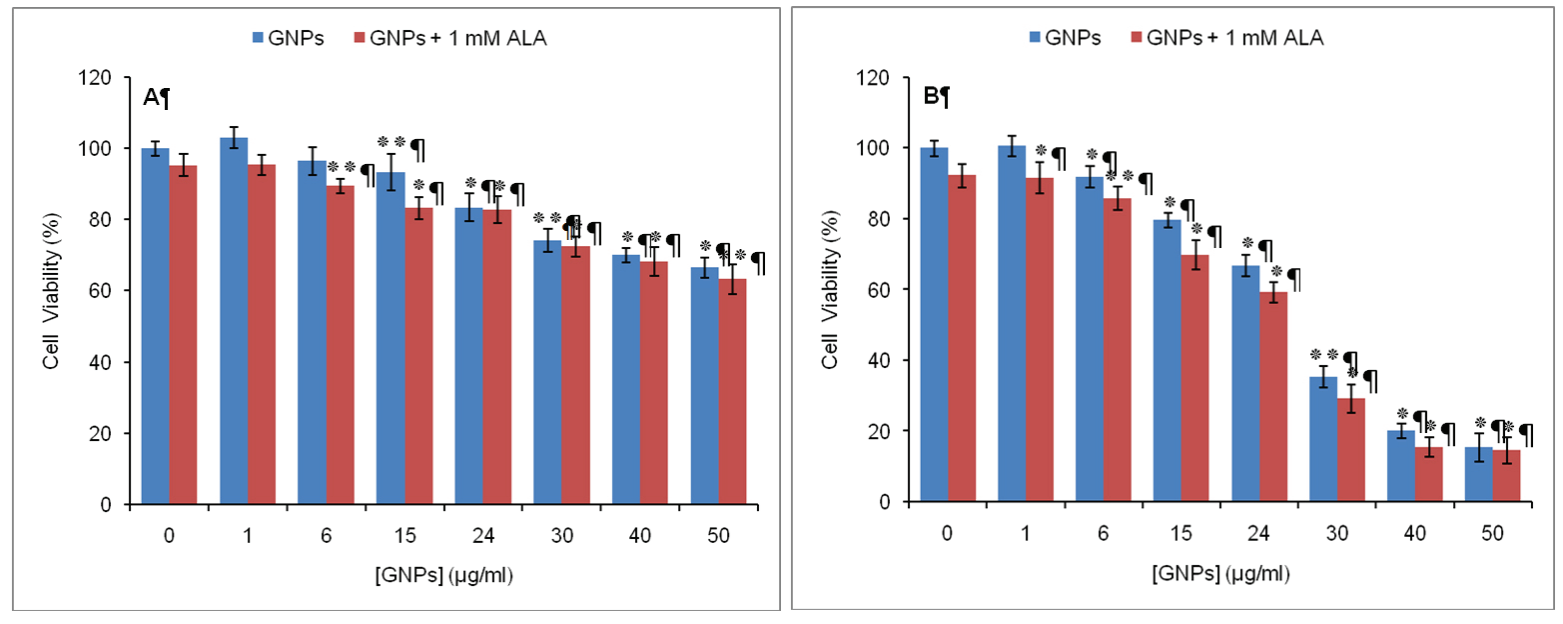
Dark cytotoxic effects on human fibroblast cells (A) and A431 cells (B) incubated in the simultaneous presence of 5-ALA and GNPs. Viability for each cell line was referenced to that of the control sample incubated with medium containing neither 5-ALA nor GNPs. Data are the mean±SD of three independent experiments (n=5). **P<0.01, *P<0.05 vs. control

As shown in figure 3A, the percentage of cell viability was above 80% for fibroblast cells incubated in the dark with 1 mM 5-ALA plus GNPs at 1, 6, 15 and 24 μg/ml concentrations. As the concentration of GNPs increased from 30, 40 to 50 μg/ml, cell viability decreased from 72%, 68% to 63% respectively.

In A431 cells, viability remained high (>85%) at low concentrations of GNPs (<15μg/ml). However, in the presence of higher concentrations of GNPs with 1 mM 5-ALA, A431 cells were found to be more sensitive than normal fibroblasts such that at 15, 24, 30, 40 and 50 μg/ml of GNPs, cell viability decreased to 70%, 59%, 29%, 15% and 14% respectively (figure 3B).

*5-ALA-Mediated Photodynamic Therapy*

**Figure 4:**
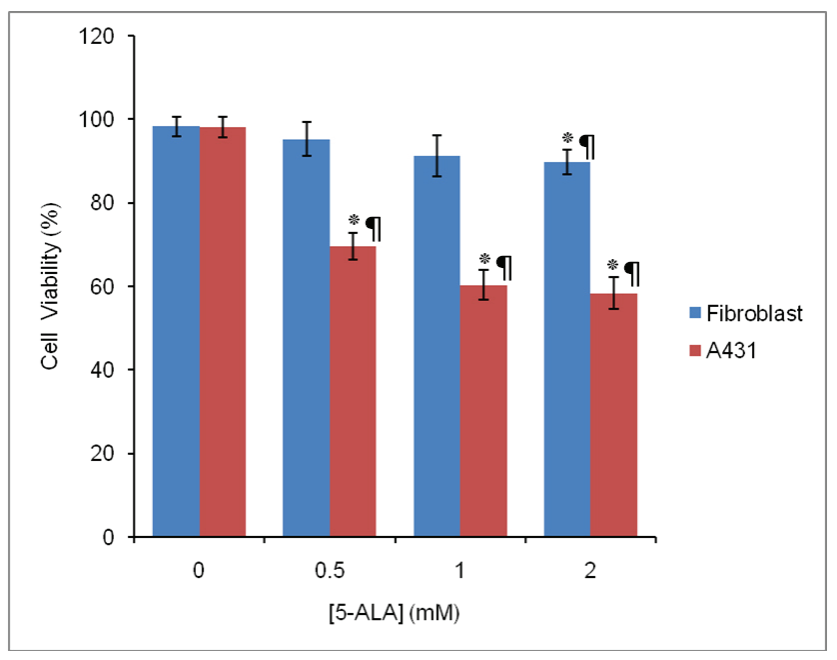
Comparison of the cell viability (%) of fibroblast and A431 cells treated with various concentrations of 5-ALA and light dose treatment of 25 J/cm^2^(5-ALA-PDT). Data are the mean±SD of three independent experiments (n=5). **P<0.01, * P<0.05 vs. control

As shown in figure 4, photoirradiation of fibroblast cells (pre-incubated with different concentrations of 5-ALA alone for 4h, using 25 J/cm^2^) caused a slight decrease (5-10%) in the viability of cells. In contrast, PDT mediated by 5-ALA was effective for A431 cell line, as it resulted in 32%, 38% and 45% cell death in the presence of 0.5, 1 and 2 mM 5-ALA respectively.

*Effect of GNPs on 5-ALA-Mediated Photodynamic Therapy*

To evaluate the effect of GNPs on the 5-ALA-PDT efficacy, phototoxicity of 5-ALA in the presence of GNPs in A431 cells was assessed by the MTT assay and compared with the effects of each of them alone.

GNPs concentrations that were minimally toxic in the absence or presence of light, were chosen for further analysis in combination with 5-ALA. Thereafter, A431 cells incubated with these low toxic concentrations of GNPs (1, 6, 15 and 24 μg/ml) plus 1 mM 5-ALA and subsequently illuminated with 25 J/ cm^2^ as described previously.

**Figure 5:**
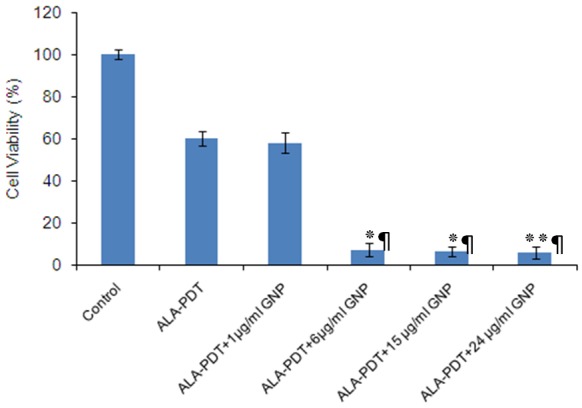
The effect of ALA-PDT alone and in combination with different GNPs concentrations on cell viability of A431 cells. Data are the mean±SD of three independent experiments (n=5). **P<0.01, *P<0.05 vs. ALA-PDT alone

As shown in figure 5, GNPs at a constant concentration of 1 μg/ml had no effect on the results of 5-ALA- PDT, but at concentrations of 6, 15 and 24 μg/ml, it resulted in a decrease of cellular survival to approximately six-fold more than 5-ALA alone. A 90-95% cell death was measured with 1 mM 5-ALA plus GNPs at concentrations of 6, 15 or 24 μg/ml. Overall, these in vitro results shows a synergistic inhibitory effect on viability of the A431 cells after combination of 5-ALA with the indicated concentration of GNPs.

## Discussion

Previously It has been shown that the 5-ALA/GNP conjugates have the potential for selective delivery of 5-ALA to tumor cells and enhanced PDT efficacy. Studies from Oo et al.^24^ demonstrated that 5-ALA-conjugated nanoparticles result in 50% more cytotoxicity to human fibrosarcoma cells than that of 5-ALA alone. In a study done by Xu et al.^25^ 5-ALA-GNP conjugates showed greater cytotoxicity against K562 cells than 5-ALA alone. However, as much as known, no studies about the effect of GNPs as a single agent and without any specific functionalization on the efficacy of 5-ALA-PDT in A431 cells have been reported. Therefore, this study aimed at exploring whether GNPs (even in the absence of any specific functionalization) can induce an increase in cell death during PDT. For this purpose, epidermoid carcinoma cells (A431) as a prototype of skin cancer cells and the human fibroblasts as normal cells were used.

In this study, it is demonstrated that in the A431 cell line, there is a synergistic effect on the inhibition of cell growth between 5-ALA-PDT and GNPs.

Although GNPs are known to be biocompatible at particular concentrations, there are reports on their toxicity in some cell lines.^26^ It was therefore necessary to determine the maximum concentration of GNPs in which they exhibit acceptable biocompatibility. The results show that, dark and phototoxicity of GNPs depends on the concentration of nanoparticle and cell type. GNPs at low concentrations (<30 μg/mL) had little toxicity to A431 and fibroblast cells. At higher concentrations (≥30 μg/mL), GNPs resulted in a dose-dependent decrease in cell viability of A431 and fibroblast cells. However, A431 cells were more sensitive to GNPs than fibroblasts. The results of this investigation were in good agreement with recent study reports. Zhang et al. reported that high concentration of GNPs (>150 mg/mL) could cause a sharp decrease in K562 cell viability, while low concentration of GNPs (<75 μg/mL) had no obvious influence on cell viability.^27^ Coulter et al. showed that 1.9 nm GNPs eradicate cancer cells (human DU145 prostate cancer and MDA-MB-231 breast cancer cells) in a dose and time dependent manner, while nontoxic to normal cells (L132 lung epithelial cells).^28^ Although the exact mechanism of cell death is not determined in the present experiments, it is reported that GNPs with diameters of about 5 nm or less are catalytically active for reduction of molecular oxygen.^29^ Thus, it seems that dark and phototoxicity of GNPs for A431 and fibroblast cells is related to the increased production of ROS and oxidative damage to cellular components such as proteins, lipids, and DNA.

In the present study, 5-ALA-induced toxicity in the absence and the presence of light against both cell lines is also evaluated. It is shown that there is none or only little dark toxicity after incubation cells with 5-ALA alone, while 5-ALA was toxic to cells after being activated by red light. Low dark toxicity of 5-ALA is reported by other researchers. Studies done by Berlanda et al. shows that 5-ALA at concentrations lower than 3 mM has no dark cytotoxic effects on A431 cells.^30^ Battah et al. also shows that dark toxicity of 5-ALA is less than 10% in A431 cells.^31^ On the other hand, current phototoxicity study shows that A431 cells are much more sensitive to 5-ALA-PDT than normal fibroblast cells. Low dark toxicity and high selectivity for cancer cells is among the important features of a good photosensitizer. Based on the results obtained in this study, 5-ALA can therefore be considered as a suitable photosensitizer for PDT against A431 cells.

Furthermore, this study also demonstrates that 5-ALA-PDT in combination with GNPs, results in a synergistic reduction in viability of A431cells. The mechanism by which GNPs act synergistically with 5-ALA in promoting 5-ALA-PDT is unclear. However, given that the effectiveness of PDT depends on the production of ROS within the cell, it seems that such synergistic effect is caused by the accumulation of GNPs in A431 cells. Thus, more ROS is produced in cells via the strongly localized electric field of GNPs.

Future research work would aim at determining the exact molecular mechanism of such synergistic action.

## Conclusion

Herein, it is shown that GNPs in combination with 5-ALA could be useful in improving the effectiveness of 5-ALA-PDT in the human epidermoid carcinoma cells. However, the exact molecular mechanism of such synergistic action remains to be determined.
